# Expression analysis of SOX2 and SOX9 in patients with oral squamous cell carcinoma

**DOI:** 10.1002/hed.27925

**Published:** 2024-08-23

**Authors:** Sonja Steen, Dominik Horn, Christa Flechtenmacher, Jürgen Hoffmann, Kolja Freier, Oliver Ristow, Jochen Hess, Julius Moratin

**Affiliations:** ^1^ Department of Oral and Cranio‐Maxillofacial Surgery University of Heidelberg Heidelberg Germany; ^2^ Department of Oral and Cranio‐Maxillofacial Surgery Saarland University Hospital Homburg Germany; ^3^ Tissue Bank of the National Center for Tumor Diseases (NCT) Heidelberg Germany; ^4^ Institute of Pathology University of Heidelberg Heidelberg Germany; ^5^ Department of Otorhinolaryngology – Head and Neck Surgery University of Heidelberg Heidelberg Germany

**Keywords:** oral cancer, OSCC, SOX2, SOX9, survival

## Abstract

**Background:**

Lately SOX2 and SOX9, transcription factors associated with stemness‐like phenotypes of cancer cells, have been linked to tumor growth, metastasis, and resistance to therapy.

**Methods:**

This study aimed on evaluating the expression of SOX2 and SOX9 in a large cohort of patients with OSCC including primary and recurrent tumors and corresponding lymph node metastases. Semiautomatic digital pathology scoring was used to determine protein expression and survival analysis was performed to evaluate its prognostic significance.

**Results:**

We found a significant downregulation of SOX9 from primary disease to lymph node metastases (*p* < 0.001). SOX9 expression and the subgroup SOX2^low^SOX9^high^ were significantly correlated with worse overall survival (*p* < 0.05). Additionally, SOX2^low^SOX9^high^ expression pattern was confirmed as independent prognosticator for overall survival.

**Conclusions:**

These results indicate the relevant role of SOX2 and SOX9 in patients with OSCC and show the clinical relevance for further investigation on the molecular mechanisms underlying SOX‐related gene expression.

## 1 INTRODUCTION

Head and neck cancer as the seventh most common group of malignant tumors afflicts about 900 000 new cases per year and a major part of these tumors are diagnosed as oral squamous cell carcinoma (OSCC).^1^, ^2^, ^3^ They show an aggressive growth pattern with a high degree of local invasiveness,^4^, ^5^ and 26%–80% of patients subsequently develop locoregional recurrence or distant metastases.^6^ Despite significant improvements in surgery, radio‐ and chemotherapy (R(C)T), long‐term survival rates in patients with advanced stage of OSCC have not significantly increased in the past 30 years.^7^, ^8^ Looking at the main causes of treatment failure, the main contributor to low patient survival is R(C)T resistance leading to tumor‐relapse and been attributed to stemness‐like features of cancer cells.^9^, ^10^

Cancer stem‐like cells (CSCs) exhibit diverse cell properties including self‐renewal, differentiation capacity, and resistance to apoptosis and are responsible for tumor maintenance and metastasis.^11^, ^12^ Furthermore, they are also responsible for resistance towards (R(C)T).^12^, ^13^ It is therefore of major importance to investigate the influence of stem cell transcription factors, including SOX2 and SOX9, that have been linked to CSC regulation, as they may be important on clinical outcome of patients with OSCC.^14^, ^15^ SOX2 and SOX9 are frequently overexpressed in multiple cancer entities, suggesting a link between malignancy and stemness.

Sex‐determining region Y (SRY)‐box 2 protein (SOX2) is expressed in CSCs and it has been used widely as a marker to identify CSCs. Many studies have proven that SOX2 contributes to carcinogenesis, such as skin squamous cell carcinoma, gastric cancer, glioblastoma, colorectal cancer, lung cancer, breast cancer, and HNSCC.^16^, ^17^, ^18^, ^19^, ^20^, ^21^, ^22^ Moreover, SOX2 has been described as a prognostic factor for different types of cancers, although there are reports on an association with both, good and bad outcome, depending on the context of tumor entity and stage of disease.^19^, ^23^ Sex‐determining region Y (SRY)‐box 2 (SOX2) and ‐box 9 protein (SOX9) are members of the high‐mobility group (HMG)‐box class of transcription factors. They control critical steps in embryonic development, stem cell homeostasis, and differentiation.^24^, ^25^, ^26^ On one hand SOX9 has been implicated as an oncogene in different types of cancer (e.g., esophageal squamous cell carcinoma, breast and colorectal cancers) and its adaptable role to participate in different steps of cancer progression, and on the other hand findings suggest that it may also behave as a tumor suppressor (e.g., cervical cancer).^27^, ^28^, ^29^, ^30^ Furthermore, SOX9 has been linked to radiotherapy resistance in HNSCC and to resistance to cisplatin therapy in non‐small cell lung cancer.^10^, ^31^

So far, the knowledge on SOX2 and SOX9 regulation and function in OSCC is limited, and the impact of both transcription factors on the clinical patient outcome remains controversial.^32^, ^33^, ^34^ Up to date there has not been an investigation of SOX2 and SOX9 expression in tissue samples of neck node metastases and recurrent tumors in patients with OSCC. Therefore, the main objective of this study was to evaluate the expression of SOX2 and SOX9 in tissue samples of a homogenously surgically treated cohort of 222 patients with OSCC, 75 matched lymph node metastases and 33 matched recurrent tumors via immunohistochemistry. We investigated the dynamics of SOX2 and SOX9 expression in primary tumors, neck node metastases, and recurrent tumors and evaluated the association with clinical and pathological parameters and survival data.

## 2 MATERIALS AND METHODS

### 2.1 Patients and samples

The investigated cohort consisted of 222 patients with primary OSCC (Table 1), 75 matched lymph node metastasis and 33 recurrent tumors. All patients were surgically treated at the Department of Oral and Cranio‐Maxillofacial Surgery of the University Hospital Heidelberg between 2010 and 2017 and were retrospectively collected. In case of residual disease, lymph node metastases or histopathological risk factors additional adjuvant radiation or systemic therapy was applied. All experimental procedures were approved by the Ethics Committee of the Medical Faculty of the University of Heidelberg (Ethic vote: S‐360/2011) and written informed consent was obtained from all patients. This study was conducted in accordance with the Declaration of Helsinki. Clinical and therapeutic data was assessed via SAP patient management research (SAP, Walldorf, Germany).

**TABLE 1:** Demographic, clinical, and pathological data of the investigated cohort.

Parameter	Number of cases	Percentage distributions of cases
Sex		
Female	85	38.3%
Male	137	61.7%
Age		
<64 years	107	48.2%
>64 years	115	51.8%
T classification		
T1	82	36.9%
T2	72	32.4%
T3	8	3.6%
T4	60	27.0%
N classification		
N0	147	66.2%
N1	27	12.2%
N2a	1	0.5%
N2b	28	12.5%
N2c	18	8.1%
N3	1	0.5%
M classification		
M0	222	100%
M+	0	0.0%
Grading		
G1	17	7.7%
G2	153	68.9%
G3	46	20.7%
Missing	6	2.7%
UICC		
Stadium I	69	31.1%
Stadium II	44	19.8%
Stadium III	23	10.4%
Stadium IV	86	38.7%
Resection status		
Complete resection (R0)	210	94.6%
Incomplete resection (R1)	10	4.5%
Missing	2	0.9%
Localization		
Floor of the mouth	64	28.8%
Tongue	54	24.3%
Mandible	70	31.5%
Maxilla	5	2.3%
Lower lip	1	0.5%
Oropharynx	14	6.3%
Buccal plane	14	6.3%
Disease recurrence		
Yes	39	17.6%
No	183	82.4%
Recurrence classification		
Local recurrence	27	69.2%
Cervical metastases	11	28.2%
Distant metastases	1	2.6%
Adjuvant therapy		
No adjuvant therapy	133	59.9%
Radiotherapy	54	24.3%
Radiochemotherapy	31	14.0%
Radioimmunotherapy	4	1.8%
Pathologic risk factors		
Perineural infiltration (Pn)	5	2.3%
Lymphatic infiltration (L)	17	7.7%
Vascular infiltration (V)	3	1.4%
Extracapsular spread (Ecs)	30	13.5%

### 2.2 Tissue microarray and histological slices

The tissue samples were provided by the Tissue Bank of the National Center for Tumor Diseases (NCT) Heidelberg, Germany in accordance with the regulations of the tissue bank and the approval of the ethics committee of Heidelberg University. The tissue microarrays (TMAs) and histological slices were prepared according to an established protocol by the tissue bank of the National Center for Tumor Diseases (NCT) Heidelberg, Germany, which has been previously described.^35^ After the preselection and marking of the representative tissue areas by an experienced pathologist, tissue cores were extracted from the paraffin blocks via the tissue chip microarray (Beecher Instruments, Sun Prairie, WI) and then transferred into recipient paraffin blocks. Two representative tissue cores (1.2 mm diameter) were taken from each tumor block to construct TMAs of primary OSCCs and TMAs of the matched lymph node metastases. Slides with a thickness of 2–3 μm were produced for the staining procedure (Histo Bond, Marienfeld, Germany). Representative tumor areas of patients with recurrent disease were generated as whole histological paraffin slices.

### 2.3 Immunohistochemistry

All TMAs and histological slices were stained using anti‐SOX2 (D6D9) (#3579, Cell Signaling Technology, Danvers, MA) and anti‐SOX9 (3C10) (ab76997, Abcam, Cambridge, UK) monoclonal antibodies following the manufacturer's instruction and using ImPRESS Reagent detection system (Vector Laboratories, Inc., Burlingame, CA) and DAB chromogen as substrate (Vector Laboratories, Inc., Burlingame, CA). All stained slides and TMAs were scanned with the Ventana DP200 slide scanner (Roche Holding AG, Basel, Switzerland).

Finally, the patients were subdivided into low and high expression groups based on their calculated scores (SOX2: low expression group: H‐Score 0–11, high expression group: H‐Score 12–213; SOX9: low expression group: H‐Score 0–64, high expression group: H‐Score 65–151).

### 2.4 Digital pathology‐based scoring

**FIGURE 1:**
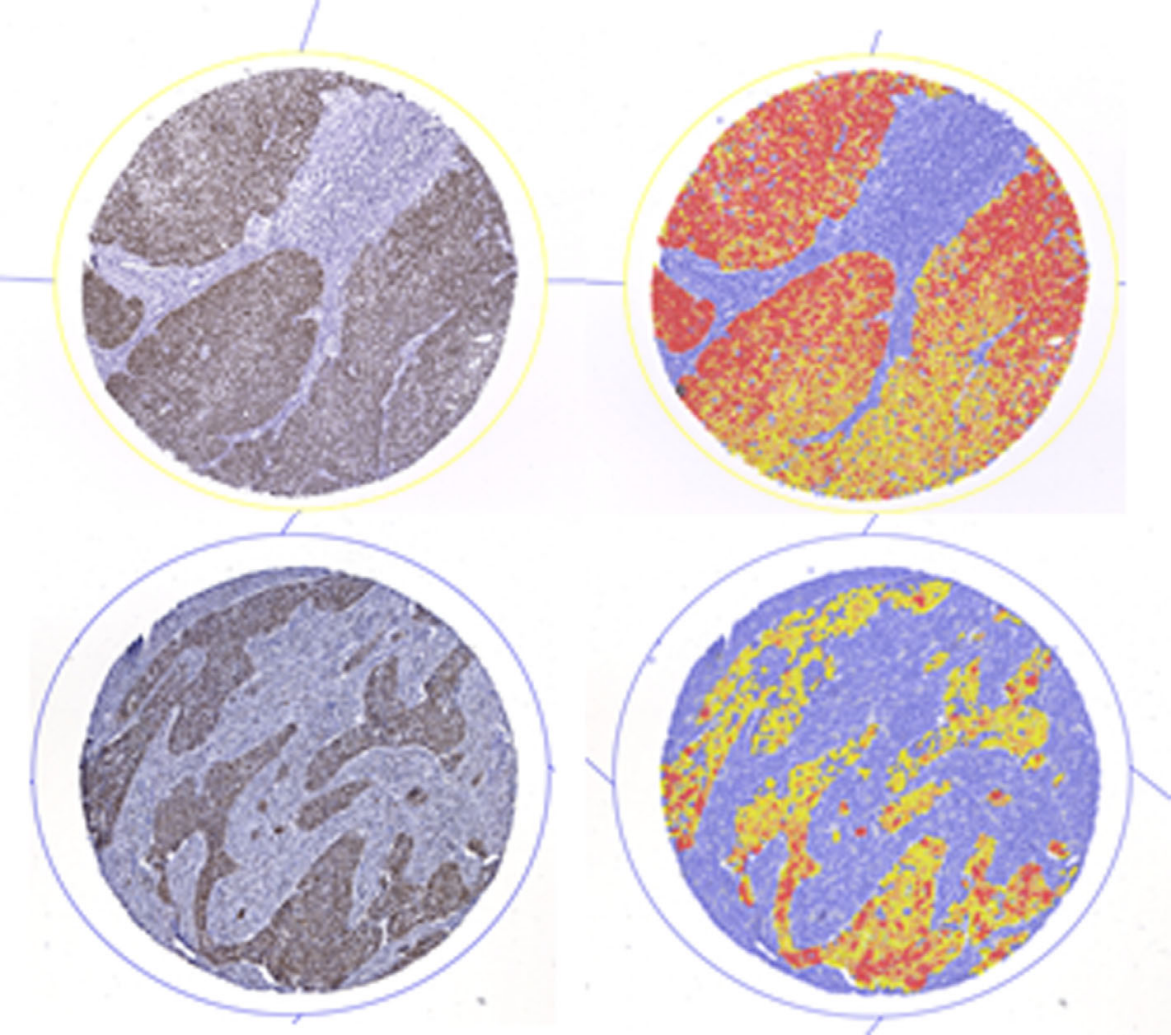
Expression analysis using QuPath. Expression analysis of SOX2 and SOX9: analysis using QuPath with and without displaying the positive cell detection. (A) Expression of SOX2; (b) expression of SOX9. [Color figure can be viewed at wileyonlinelibrary.com]

Qupath version v0.2.3 was used for semi‐automatic quantification of the immunohistochemical stains. In the first step, the TMA dearrayer function was used to infer the TMA grid. Invalid samples or artifact regions were manually excluded. In the next step the stain vectors were automatically determined for every TMA and histological slide. Next, the positive cell detection function was used to quantify the number of positive cells and by setting an individual threshold for each antibody. The staining intensity was assessed, dividing the positive staining intensity in none, low, moderate, and high. For every antibody the optimal score compartment was determined (nucleus means for SOX2 and cell means for SOX9). Finally, an H‐Score was generated by QuPath, representing a score, which is composed by the percentage of nuclei and the staining intensity. Figure 1 shows examples of the semi‐automatic scoring of SOX2 and SOX9 on TMAs.

In contrast to the TMAs, in the scoring procedure of whole histological slides, another step was added. By using a detection classifier, it was possible to distinguish different tissues and to set them into specific classes. Five different classes were set up by annotating representative cells for each class (e.g., tumor, stroma, and immune cells). This made it possible to consider only the positive staining in the tumor.

### 2.5 Statistical analysis

Statistical analyses were performed using Microsoft Excel (Microsoft, Redmond, WA) and SPSS 27 (SPSS for Mac, SPSS, Chicago, IL). SOX2 and SOX9 staining scores were classified on the basis of values below or above a certain H‐Score as high expression or low expression groups. The H‐Score was calculated for each tissue core, reflecting the overall percentage of positive cells and the staining intensity, giving results in the range 0 (all tumor cells negative) to 300 (all tumor cells strongly positive).^36^

Bivariate analysis by chi‐square testing and cross‐tabulation were used to determine associations between SOX2 and SOX9 expression scores including their different subgroups and clinical data. The correlations between the different proteins in primary OSCC and lymph node metastases tissues were analyzed by Spearman's rank correlation test. Survival analysis was carried out using the Kaplan–Meier method from date of diagnosis until death, disease recurrence or end of data collection. Log‐rank‐testing was used to determine the statistically significant differences between the groups. Univariate and multivariate Cox regression models were applied to evaluate the impact of SOX2 and SOX9 protein expression and their subgroups on overall survival and progression‐free survival, using clinicopathological variables as covariates. The multivariate analysis was considered separately based on the dependent parameters of SOX9 expression and its subgroup, as well as the dependent parameters lymph node metastasis and extracapsular spread. *p*‐values less than 0.05 were considered statistically significant.

## 3 RESULTS

### 3.1 Patient cohort

A total of 222 patients were included in the analysis. Hundred and thirty‐seven patients (61.7%) were male, 85 (38.3%) were female. The ages ranged from 27 to 88 years with a mean age of 64.3 ± 11.1 years. Patients suffering from primary and recurrent squamous cell carcinoma of the oral cavity received between 2010 and 2017 surgical treatment in the Department of Oral and Cranio‐Maxillofacial Surgery of the University of Heidelberg. Furthermore, adjuvant radiotherapy or radio‐chemotherapy (RCT) was applied in patients with advanced tumor stage (Stage III/IV), incomplete tumor resection (R+) or the presence of histopathological risk factors, such as perineural (PN+), lymphatic (L+), or vascular (V+) tumor infiltration. An overview of the most relevant demographic, clinical and pathological features of the patient cohort provides Table 1.

### 3.2 SOX2 and SOX9 expression in primary OSCC

**FIGURE 2:**
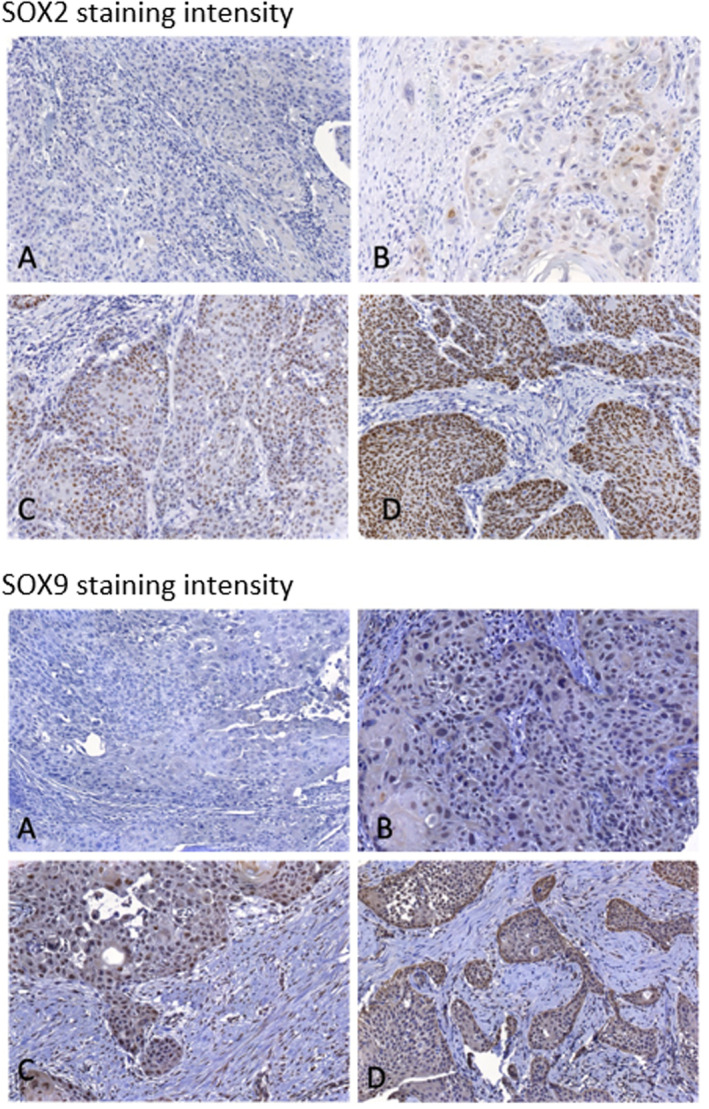
Staining intensities of SOX2 and SOX9. Immunohistochemistry staining of SOX2 and SOX9 expression in the primary oral squamous cell carcinomas (OSCC) on tissue microarrays (TMA) (A: no expression, B: low expression, C: intermediate expression, D: high expression). [Color figure can be viewed at wileyonlinelibrary.com]

Overall 222 primary tumors were revised for SOX2 and SOX9 expression by IHC. The mean H‐Score for SOX2 in primary tumors was 26.47 ± 40.18 and for SOX9 46.40 ± 32.23. Concerning sex, there was a significant higher SOX2 expression in male patients (mean SOX2 scores: male patients 30.88 ± 43.90, female patients 19.35 ± 32.33, *p* = 0.046). No significant difference was detected between male and female patients for SOX9 expression (mean SOX9 scores: male patients: 46.64 ± 31.14, female patients: 46.02 ± 34.07, *p* = 0.893). Figure 2 exemplifies tissue samples incubated with either anti‐SOX2 or anti‐SOX9 antibodies representing different staining intensities.

### 3.3 SOX2 and SOX9 expression in lymph node metastases

A total number of *n* = 75 lymph node metastases was analyzed. The mean H‐Score for SOX2 in neck node metastases was 13.67 ± 28.48 and for SOX9 20.93 ± 31.32. There was no significant difference between the SOX2 and SOX9 expression in male and female patients in lymph node metastases (mean SOX2 scores: male patients: 14.93 ± 30.77, female patients: 11.17 ± 23.80, *p* = 0.643; mean SOX9 scores: male patients: 22.026 ± 32.65, female patients: 18.69 ± 29.10, *p* = 0.707).

### 3.4 SOX2 and SOX9 expression in recurrent tumors

Thirty‐three recurrent tumors were revised for SOX2 and SOX9 expression by IHC. The mean H‐Score for SOX2 in recurrent tumors was 98.04 ± 83.31 and for SOX9 156.24 ± 56.09. There was no significant difference between recurrent tumors from male and female patients for SOX2 and SOX9 expression (mean SOX2 scores: male patients: 109.48 ± 83.44, female patients: 78.01 ± 82.74, *p* = 0.304; mean SOX9 scores: male patients: 150.86 ± 58.04, female patients: 165.67 ± 53.62, *p* = 0.474).

Tables 2 and 3 provide an overview of the different H‐Scores of SOX2 and SOX9 expression in primary tumors, lymph node metastases and recurrent tumors.

**TABLE 2:** Overview of mean values of SOX2 in primary tumors, lymph node metastases, and recurrent tumors.

	Average	Median	Minimum	Maximum	Standard deviation	*N*
Primary tumors	26.47	6.61	0.02	213.50	40.18	204
Lymph node metastases	13.67	1.26	0.01	153.82	28.48	57
Recurrent tumors	98.06	66.46	0.19	233.29	83.31	33

**TABLE 3:** Overview of mean values of SOX9 in primary tumors, lymph node metastases, and recurrent tumors.

	Average	Median	Minimum	Maximum	Standard deviation	*N*
Primary tumors	46.40	44.95	0.04	151.50	32.23	209
Lymph node metastases	20.93	7.12	0.00	137.14	31.32	58
Recurrent tumors	156.24	176.00	22.00	229.00	56.09	33

### 3.5 Comparison between SOX2 and SOX9 expression in primary tumors, in lymph node metastases and in recurrent tumors

The scores of 45 lymph nodes metastases were compared to those of their corresponding primary tumors. The H‐Score of the primary tumor samples and the lymph node metastases differed significantly with lower scores in lymph node metastases for SOX9 (paired *t* test: *p* < 0.001). For SOX2, there was no significant difference.

**FIGURE 3:**
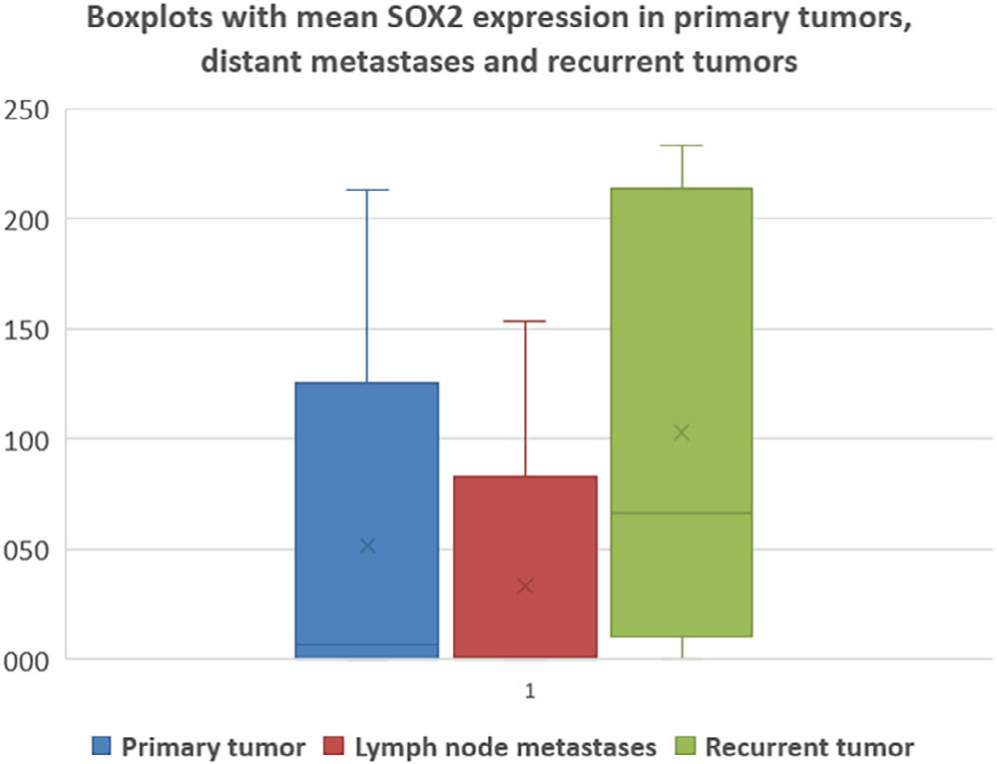
Mean SOX2 expression in primary tumor tissue, lymph node metastases, and recurrent tumors. [Color figure can be viewed at wileyonlinelibrary.com]

**FIGURE 4:**
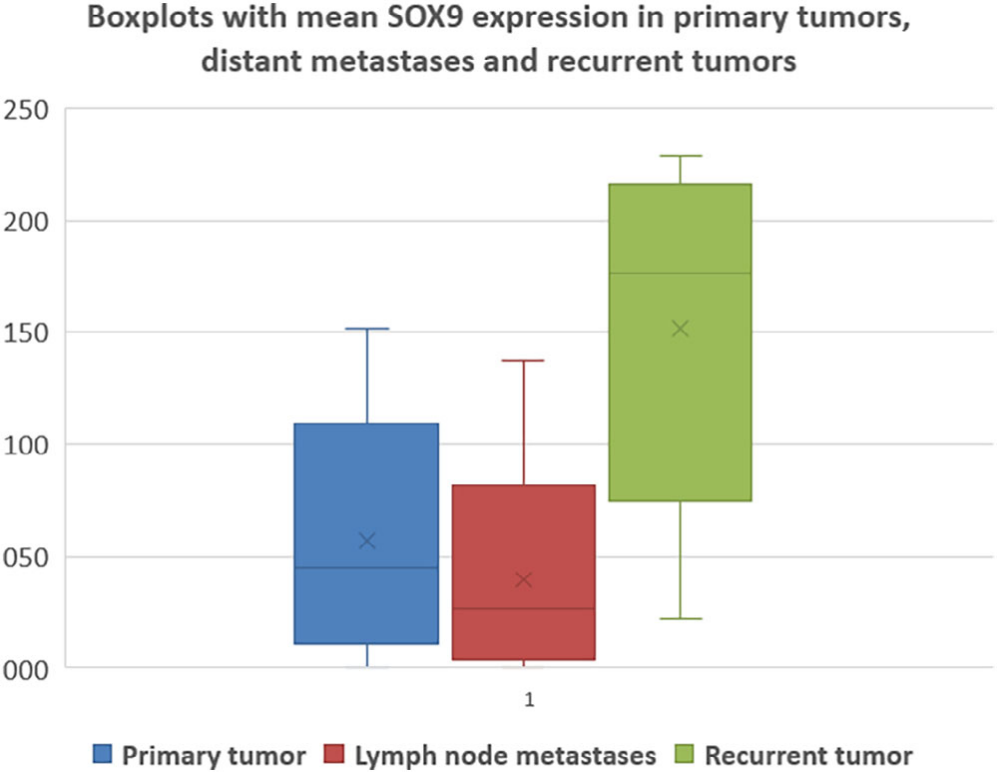
Mean SOX9 expression in primary tumor tissue, lymph node metastases, and recurrent tumors. [Color figure can be viewed at wileyonlinelibrary.com]

The comparison of SOX2 and SOX9 expression levels in primary and recurrent tumors revealed a significant increase in recurrent diseases (SOX2 paired *t* test *p* < 0.001; SOX9 paired *t* test *p* < 0.001). In addition, a significant increase was observed for SOX2 and SOX9 expression in recurrent tumors compared to lymph node metastases (SOX2 paired *t* test *p* < 0.001; SOX9 paired *t* test *p* < 0.001). An overview of the expression levels for SOX2 and SOX9 in primary tumors versus corresponding lymph node metastases and recurrent tumors are shown in Table 4. The graphical representation of the expression of SOX2 and SOX9 in primary tumors, lymph node metastases, and recurrences are shown in Figures 3 and 4.

**TABLE 4:** SOX2 and SOX9 expression in primary tumors versus corresponding lymph node metastases and recurrent tumors.

	Average	Differences in mean values	Standard deviation	*N*	Significance (2‐sided)
SOX2 in primary tumors vs. LNM	20.88 14.27	6.61	30.04	45	0.147
SOX2 in primary tumors vs. recurrent tumors	24.41 103.38	−78.98	66.32	27	<0.001
SOX2 in LNM vs. recurrent tumors	8.94 91.93	−83.00	78.72	15	0.001
SOX9 in primary tumors vs. LNM	47.52 20.30	27.22	49.01	47	<0.001
SOX9 in primary tumors vs. recurrent tumors	56.23 154.90	−98.66	58.25	29	<0.001
SOX9 in LNM vs. recurrent tumors	15.77 142.93	−127.16	56.50	15	<0.001

No significant difference was detected for SOX2 and SOX9 expression levels between: (i) patients with recurrence and with or without prior adjuvant therapy (SOX2 H‐Score with adjuvant therapy 90.30 ± 79.14, SOX2 H‐Score without adjuvant therapy: 104.48 ± 88.38; *p* = 0.634 and SOX9 with adjuvant therapy 141.27 ± 67.53, SOX9 H‐Score without adjuvant therapy 168.73 ± 42.4; *p* = 0.165), or (ii) primary tumors of patients with or without later recurrence (patients without recurrence: SOX2 H‐Score: 26.02 ± 40.43, SOX9 H‐Score: 44.99 ± 31.49 and patients with recurrence: SOX2 H‐Score: 32.31 ± 39.80, SOX9 H‐Score: 54.69 ± 34.61) (SOX2 *t* test, *p* = 0.41, SOX9 *t* test = 0.10).

### 3.6 Association of SOX2 and SOX9 expression with clinicopathological variables

The 222 tumors were categorized into two groups using the best cut‐off regarding overall survival of ≥12 (H‐Score) for SOX2 and ≥65 (H‐Score) for SOX9. There was a statistically significant correlation between SOX2 and the age (64 years, *p* < 0.025), the pathological tumor size (T1/T2 vs. T3/T4, *p* < 0.001), the clinical stage (I/II vs. III/IV, *p* < 0.004), and extracapsular spread (Ecs0 vs. Ecs+, *p* < 0.031). However, there was no significant difference for SOX9 and any clinicopathological characteristics tested.

Looking at the subgroups, SOX2^high^SOX9^low^ significant correlation between this subgroup and the tumor size (*p* < 0.042), lymph node metastases (*p* < 0.021), clinical stage (*p* < 0.014), and sex (*p* < 0.021) can be found. Between the subgroup SOX2^low^SOX9^high^ there is a significant correlation between this subgroup and the tumor size (*p* < 0.006). Furthermore, we found a significant correlation between the subgroup SOX2^low^SOX9^low^ and the the tumor size (*p* < 0.026), lymphatic infiltration (*p* < 0.012), and extracapsular spread (*p* < 0.006). For other clinicopathological characteristics there was no significant difference. The results of the correlation analysis are shown in Tables 5 and 6.

**TABLE 5:** Correlation of clinical and histopathological parameters.

	SOX2^low^ (*N*)	SOX2^high^ (*N*)	*p*‐value	SOX9^low^ (*N*)	SOX9^high^ (*N*)	*p*‐value
Age						
<64 years	48	47	**0.025**	65	32	0.260
>64 years	72	37		83	29	
Sex						
Male	69	57	0.134	94	34	0.294
Female	51	27		54	27	
T classification						
T1/T2	73	72	**0.000**	103	45	0.546
T3/T4	47	12		45	16	
N classification						
Node negative	79	63	0.161	105	40	0.444
Node positive	41	21		43	21	
Stage						
I/II	54	55	**0.004**	79	32	0.904
III/IV	66	29		69	29	
Grading						
G1	9	8	0.652	13	4	0.821
G2	79	57		99	42	
G3	29	16		33	12	
Perineural infiltration						
Pn0	117	82	0.857	146	58	0.125
Pn1	3	2		2	3	
Lymphatic infiltration						
L0	108	81	0.083	135	59	0.161
L1	12	3		13	2	
Vascular infiltration						
V0	119	82	0.366	146	60	0.874
V1	1	2		2	1	
Extracapsular spread						
Ecs0	101	79	**0.031**	128	57	0.152
Ecs+	19	5		20	4	

*Note*: Significant *p*‐values printed bold.

**TABLE 6:** Correlation of clinical and histopathological parameters (subgroups).

	CG (*N*)	SOX2^low^SOX9^high^ (*N*)	*p*‐value	CG (*N*)	SOX2^high^SOX9^low^ (*N*)	*p*‐value	CG (*N*)	SOX2^high^SOX9^high^ (*N*)	*p*‐value	CG (*N*)	SOX2^low^SOX9^low^ (*N*)	*p*‐value
Age												
<64 years	86	9	0.899	71	24	0.161	72	23	0.161	56	39	0.040
>64 years	99	11		91	19		92	18		49	61	
Sex												
Male	116	10	0.267	93	33	**0.021**	102	24	0.667	67	59	0.479
Female	69	10		69	10		62	17		38	41	
T classification												
T1/T2	137	9	**0.006**	110	36	**0.042**	110	36	**0.009**	82	64	**0.026**
T3/T4	48	11		52	7		54	5		23	36	
N classification												
Node negative	129	13	0.663	106	36	**0.021**	115	27	0.596	76	66	0.322
Node positive	56	7		56	7		49	14		29	34	
UICC												
I/II	102	7	0.086	79	30	**0.014**	84	25	0.263	62	47	0.084
III/IV	83	13		83	13		80	16		43	53	
Grading												
G1	17	0	0.345	13	4	0.814	13	4	0.887	8	9	0.560
G2	122	15		107	30		110	27		73	64	
G3	41	4		37	8		37	8		20	25	
Perineural infiltration												
Pn0	181	19	0.434	157	43	0.243	161	39	0.258	102	98	0.691
Pn1	4	1		5	0		3	2		3	2	
Lymphatic infiltration												
L0	170	20	0.186	148	42	0.157	151	39	0.503	102	88	**0.012**
L1	15	0		14	1		13	2		3	12	
Vascular infiltration												
V0	182	20	0.566	160	42	0.596	162	40	0.561	103	99	0.590
V1	3	0		2	1		2	1		2	1	
Extracapsular spread												
Ecs0	162	19	0.326	140	41	0.105	143	38	0.328	99	82	**0.006**
Ecs+	23	1		22	2		21	3		6	18	

*Note*: Significant *p*‐values printed bold.

Abbreviation: CG, comparison group.

### 3.7 Survival analysis in relation to SOX2 and SOX9 expression

The mean survival time was 64.76 months (95% CI 60.88–68.64 months). No patient had distant metastasis at the time of diagnosis. Altogether 47 patients died (21.2%), while 175 (78.8%) patients remained alive at the time of follow‐up.

**FIGURE 5:**
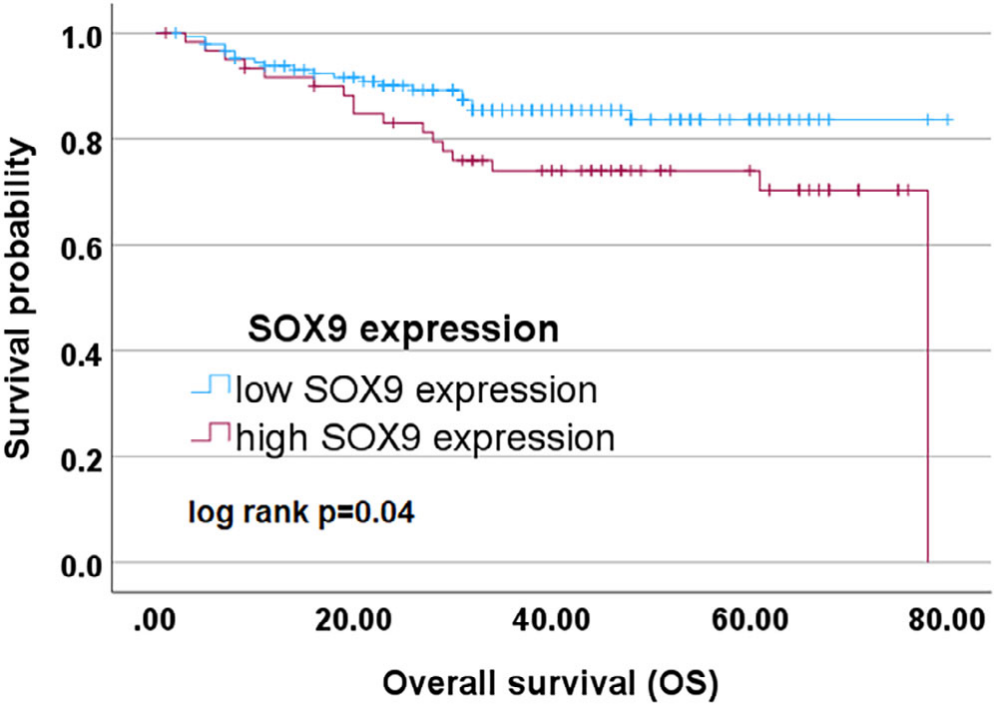
Survival analysis. Kaplan–Meier curves depicting the results of the univariate survival analysis for SOX9 status. [Color figure can be viewed at wileyonlinelibrary.com]

Kaplan–Meier analyses showed that a higher SOX9 expression in primary tumors was associated with poorer overall survival (by log‐rank test, *p* = 0.040) (Figure 5). However, there was no significant impact of SOX2 or SOX9 on progression free survival and no significant impact of SOX2 on overall survival.

**FIGURE 6:**
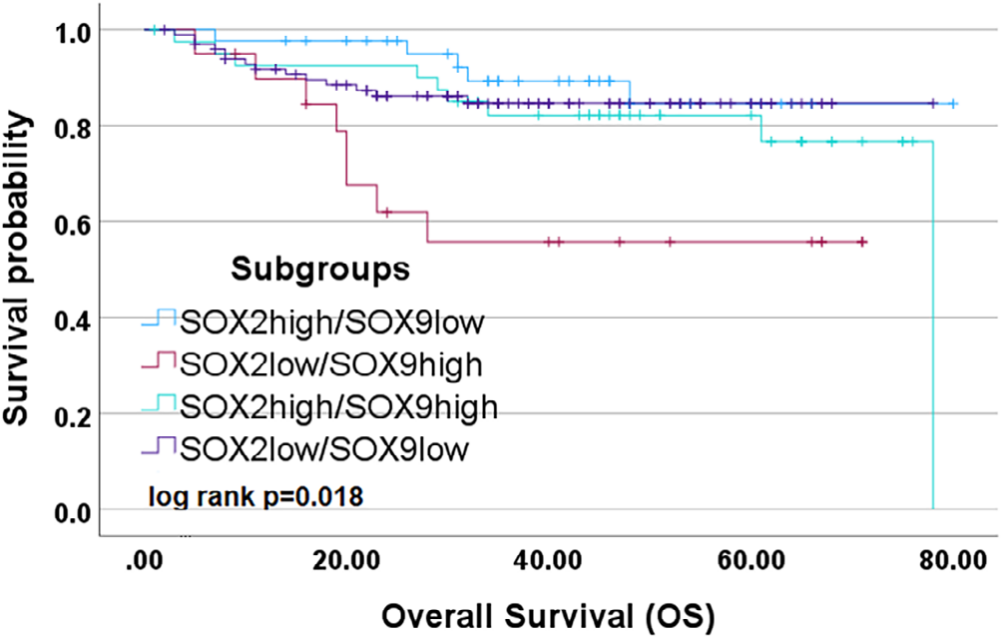
Survival analysis of subgroups. Kaplan–Meier curves depicting the results of the univariate survival analysis for the different subgroups. [Color figure can be viewed at wileyonlinelibrary.com]

**FIGURE 7:**
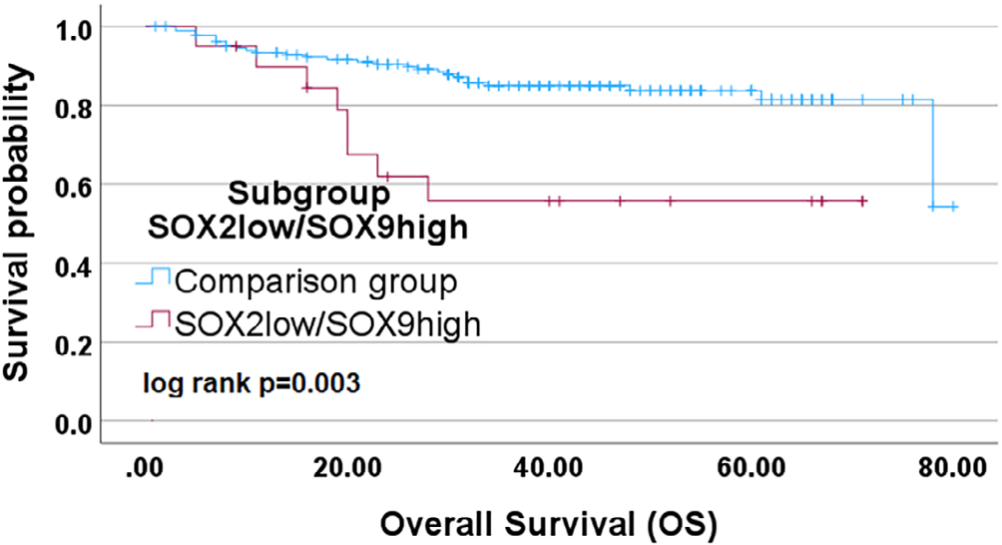
Survival analysis of the subgroup SOX2^low^SOX9^high^. Kaplan–Meier curves depicting the results of the univariate survival analysis for the subgroup SOX2^low^/SOX9^high^. [Color figure can be viewed at wileyonlinelibrary.com]

Looking at the different subgroups (SOX2^high^SOX9^high^ vs. SOX2^high^SOX9^low^ vs. SOX2^low^SOX9^high^ vs. SOX2^low^SOX9^low^) there is a clear difference in one group in terms of overall survival in the Kaplan–Meier curve. The SOX2^low^SOX9^high^ subgroup shows a significantly poorer overall survival than the other three subgroups (Figure 6). By looking at the subgroup SOX2^low^SOX9^high^ alone, the Kaplan–Meier analysis showed a strong significant impact of this group associated with poorer overall survival (*p* = 0.003) (Figure 7).

In the univariate survival analysis, we found a significant prognostic impact of SOX9 expression, the subgroup of SOX2^low^SOX9^high^ tumors, tumor size, clinical stage, nodal status, and extracapsular spread. A high SOX9 expression had a significant impact on lower overall survival (*p* = 0.044) as well as the subgroup SOX2^low^SOX9^high^ (*p* = 0.004) (Table 7). However, no significant impact was observed for SOX2, SOX9 or the subgroups for progression‐free survival (PFS). In the multivariate analysis for overall survival, the subgroup of SOX2^low^SOX9^high^ tumors, the nodal status and extracapsular spread were confirmed as independent prognostic risk factors (Table 7).

**TABLE 7:** Univariate and multivariate analysis of overall survival in consideration of relevant clinical variables and SOX2 and SOX9 protein expression.

Characteristics	Univariate	
	HR (95% CI)	*p*‐value
SOX2 expression: SOX2^high^ vs. SOX2^low^	0.747 (0.381–1466)	0.397
SOX9 expression: SOX9^high^ vs. SOX9^low^	**1.948 (1019–3725)**	**0.044**
Subgroup: SOX2^low^SOX9^high^ vs. CG	**3.158 (1431–6968)**	**0.004**
Subgroup: SOX2^high^SOX9^low^ vs. CG	0.532 (0.207–1369)	0.191
Subgroup: SOX2^high^SOX9^high^ vs. CG	1.149 (0.539–2450)	0.720
Subgroup: SOX2^low^SOX9^low^ vs. CG	0.761 (0.389–1490)	0.426
T classification: T1/T2 vs. T3/T4	**2.341 (1302–4209)**	**0.004**
N classification: N0 vs. N+	**3.568 (1990–6396)**	**0.000**
UICC stage: I/II vs. III/IV	**3.126 (1664–5874)**	**0.000**
Perineural infiltration: Pn0 vs. Pn1	1.183 (0.163–8.589)	0.868
Lymphatic infiltration: L0 vs. L1	1.811 (0.713–4.601)	0.212
Vascular infiltration: V0 vs. V1	1.441 (0.198–10.487)	0.718
Extracapsular spread: Ecs0 vs. Ecs+	**3.069 (1.584–5.948)**	**0.000**

Characteristics	Multivariate SOX9 and N classification	
	HR (95% CI)	*p*‐value
SOX9 expression: SOX9^high^ vs. SOX9^low^	1.761 (0.911–3406)	0.092
T classification: T1/T2 vs. T3/T4	**2.426 (1.008–5.836)**	**0.048**
N classification: N0 vs. N+	**3.493 (1.194–10.216)**	**0.022**
UICC stage: I/II vs. III/IV	0.639 (0.171–2.393)	0.506

Characteristics	Multivariate SOX9 and Ecs	
	HR (95% CI)	*p*‐value
SOX9 expression: SOX9^high^ vs. SOX9^low^	**1.761 (0.911–3406)**	**0.028**
T classification: T1/T2 vs. T3/T4	1.565 (0.683–3.585)	0.289
Extracapsular spread: Ecs0 vs. ECS+	**2.448 (1.032–5.806)**	**0.042**
UICC stage: I/II vs. III/IV	1.566 (0.633–3.879)	0.332

Characteristics	Multivariate subgroup and N classification	
	HR (95% CI)	*p*‐value
Subgroup: SOX2^low^SOX9^high^ vs. CG	**2.628 (1.153–5.990)**	**0.021**
T classification: T1/T2 vs. T3/T4	1.651 (0.663–4.111)	0.281
N classification: N0 vs. N+	**3.401 (1.154–10.025)**	**0.026**
UICC stage: I/II vs. III/IV	0.822 (0.215–3.147)	0.775

Characteristics	Multivariate subgroup and Ecs	
	HR (95% CI)	*p*‐value
Subgroup: SOX2^low^SOX9^high^ vs. CG	**2.995 (1.295–6.929)**	**0.010**
T classification: T1/T2 vs. T3/T4	1.040 (0.444–2.440)	0.927
Extracapsular spread: Ecs0 vs. ECS+	**2.412 (1.001–5.812)**	**0.050**
UICC stage: I/II vs. III/IV	1.982 (0.793–4.952)	0.143

*Note*: Significant *p*‐values printed bold.

## 4 DISCUSSION

In the present study, we investigated the expression of SOX2 and SOX9 in patients with primary and recurrent OSCC as well as in lymph node metastases. The role of SOX transcription factors and the effect of their dysregulation on cancer progression and on therapy response is not fully understood and some data investigating the impact of SOX2 and SOX9 in OSCC remain controversial.^4^, ^5^, ^30^, ^33^, ^37^

We found a mean SOX9 H‐Score in primary tumors of 46.40 ± 2.23 while the mean SOX9 H‐Score in lymph node metastases was 20.93 ± 4.11. The H‐Score of the primary tumor samples and the matched lymph node metastases differed significantly with lower scores in lymph node metastases for SOX9 (paired *t* test: *p* < 0.001). For SOX2 we have as well lower H‐Scores in lymph node metastases but the difference failed in significance. Furthermore, we found a concomitant gain in SOX2 and SOX9 in patients with recurrent disease (SOX2 H‐Score 26.47 ± 2.81 in primary tumors and 98.06 ± 14.50 in recurrent tumors, paired *t* test <0.001; SOX9 H‐Score 46.40 ± 2.23 in primary tumors and 156.24 ± 9.76 in recurrent tumors, paired *t* test <0.001).

Dysregulation of SOX genes in cancer have been implicated in metastasis and treatment resistance. Lin et al. emphasizes the critical involvement of epigenetic regulation between SOX2 and SOX9 in lung cancer cells.^38^ The epigenetic regulation between these two genes creates cancer plasticity between epithelial‐like and mesenchymal‐like states and endows cancer cells with different proliferative and invasive abilities.^38^ In our study patients with OSCC show lower levels of SOX2 and SOX9 in lymph node metastases while patients with tumor recurrence have significantly higher expression levels. This may indicate the importance of the morphological plasticity of cancer cells in context of tumor progression, which have to present different expression levels in order to withstand selection pressure. Lower SOX2 and SOX9 expression levels are required in lymph node metastases while high‐expression levels provide an advantage in tumor recurrence.

Furthermore, patients with primary OSCC showing a high SOX2 expression are associated with a younger age, lower tumor size, lower clinical stage, and a lower incidence of extracapsular spread. However, we did not see a clear correlation of SOX2 expression and lymph node metastases by chi‐square testing and cross‐tabulation and we found no significant impact of SOX2 on overall survival. The data regarding the correlation of SOX2 expression with clinicopathological features and the influence on overall survival among patients with OSCC remains controversial. It is one of the amplified genes in OSCC, where its expression has been closely associated with lymph node metastasis and poor prognosis.^33^, ^34^ Michifuri et al. and Qiao et al. reported that SOX2 expression in OSCC was associated with lymph node metastasis and Schröck et al. showed a tendency for lower overall survival for SOX2‐amplified patients of sinonasal carcinoma.^34^, ^39^, ^40^ In contrast, several studies implicated that high‐expression levels of SOX2 were significantly associated with lower probability of metastasis and a better prognosis in HNSCC and lung squamous cell carcinoma.^19^, ^22^, ^32^, ^41^ In patients with lung squamous cell carcinoma higher levels of SOX2 expression were as well related to younger age and smaller tumor size.^19^ The controversial results on the influence of SOX2 on clinicopathological data may indicate the heterogeneity of expression within a tumor and a context dependency.

Indeed, the subgroup of tumors with high SOX2 but low SOX9 expression (SOX2^high^SOX9^low^) was significantly associated with a smaller tumor size (*p* < 0.042) and lower probability for lymph node metastasis (*p* < 0.026). The sole consideration of SOX2 expression only showed a tendency towards less lymph node metastasis and only the analysis in conjunction with SOX9 indicated the association with lower metastasis. Therefore, additional studies on the oncogenic function of SOX2 in OSCC and its link with SOX9 accounting for the association with progression and its association to clinicopathological parameters remain to be elucidated.

In contrast to SOX2, a high SOX9 expression emerged as a risk factor for unfavorable overall survival in patients with OSCC using Kaplan–Meier analysis. Sumita et al. support this observation in a cohort of 49 patients with OSCC, where cytoplasmatic SOX9 expression correlated with poorer survival.^37^ Studies in breast cancer and non‐small cell lung carcinoma have also pointed out that SOX9 contributes to tumor progression and a poor prognosis for patients.^42^, ^43^

The subgroup analysis in our cohort demonstrated that patients with a low SOX2 expression and concomitant high SOX9 expression (subgroup SOX2^low^SOX9^high^) had a high association to a significantly poorer overall survival (*p* = 0.003). The influence of this group on overall survival is also evident in the univariate analysis (hazard ratio 3.158 and *p* = 0.004) and is also confirmed in the multivariate analysis as an independent prognostic risk factor (*p* = 0.021). When looking at SOX9 expression alone, SOX9 cannot be confirmed as an independent factor (*p* = 0.092). The relation to SOX2 expression and potential impact of epigenetic regulation thus appears to play an important role regarding the overall survival of patients with oral cavity carcinoma.

This is the first publication, to our knowledge, regarding this topic in the field of oral squamous cell carcinoma showing a group of patients with poorer overall survival by representing a distinct expression pattern of SOX2 and SOX9. The interaction between these transcription factors in the progression of cancer is complex. Multiple studies have suggested an inverse correlation between SOX2 and SOX9 expression.^38^, ^44^ Sharma et al. showed that a drug‐induced adaptation is associated with a loss of SOX2 with a simultaneously gain of SOX9 in tumor cells.^45^ In breast cancer stem cells Domenici et al. reported the maintenance of breast cancer stem cells through SOX2‐SOX9 signaling axis.^46^ In HNSCCs, Barbosa et al. demonstrated in HNSCC patients with HPV negative status that radiotherapy of tumors presenting SOX2^low^SOX9^high^ expression had a reduced survival probability when compared to the group with SOX2^high^SOX9^low^ expression.^31^ In our study, we compared the expression levels of SOX2 and SOX9 in patients with recurrence who received prior adjuvant therapy with patients with recurrence who had no prior adjuvant therapy and found no significant difference in the expression levels. However, a poorer overall survival of patients presenting SOX2^low^SOX9^high^ expression could be linked to resistance to radiotherapy and chemotherapy. So Voronkova et al. demonstrated that SOX9 overexpression in NSCLC promotes drug resistance and Barbosa et al. showed in in vitro analyses that silencing of SOX2 enhances radioresistance while SOX9 silencing enhances radiosensitivity in HNSCC.^10^, ^31^ In breast cancer an increase of SOX9 was associated with an endocrine therapy failure.^46^ Further investigations are needed to reveal the role of SOX genes in the context of treatment failure in patients with OSCC.

Recent studies underline the relevant role of SOX genes in cancer, showing that dysregulation of SOX genes in cancer have been linked to tumor progression, metastasis and treatment resistance. In our study we see variable expression levels in primary tumors, lymph node metastasis and recurrent tumors providing a link to importance of the morphological plasticity of cancer cells in the context of tumor progression, which have to present different expression levels in order to withstand selection pressure. Furthermore, we see a subgroup of inverse expression levels of SOX2 and SOX9 (SOX2^low^SOX9^high^) demonstrating a group that has a significant poorer overall survival. The association of this subgroup to poorer overall survival and radiotherapy resistance in recent literature underlines the importance to develop novel strategies for patient stratification and indicates the importance of further studies to investigate the molecular mechanisms underlying SOX‐related genes.

The presented study has several limitations. As it is a retrospective single‐center study, we cannot exclude the possibility of selection bias. The creation of subgroups of patients to assess the influence of different sets of protein expression led to small numbers of patients in several cases. This fact may impact the significance of our presented data and necessitates further validation in bigger cohorts. In addition, all our results are solely based on immunohistochemistry. Furthermore, tissue microarrays of primary tumors and lymph node metastases were compared to whole‐slide sections, which can be seen as a weakness of the study design.

## CONFLICT OF INTEREST STATEMENT

The authors declare no conflicts of interest.

## Data Availability

The data that support the findings of this study are available from the corresponding author upon reasonable request.
